# Identification of potential pathogenic targets and survival strategies of *Vibrio vulnificus* through population genomics

**DOI:** 10.3389/fcimb.2023.1254379

**Published:** 2023-08-25

**Authors:** Jia-Xin Zhang, Yuan Yuan, Qing-hua Hu, Da-zhi Jin, Yao Bai, Wen-Wen Xin, Lin Kang, Jing-Lin Wang

**Affiliations:** ^1^ State Key Laboratory of Pathogen and Biosecurity, Beijing Institute of Microbiology and Epidemiology, Academy of Military Medical Sciences (AMMS), Beijing, China; ^2^ Shenzhen Center for Disease Control and Prevention, Shenzhen, China; ^3^ Key Laboratory of Biomarkers and In Vitro Diagnosis Translation of Zhejiang Province, School of Laboratory Medicine, Hangzhou Medical College, Hangzhou, China; ^4^ China National Center for Food Safety Risk Assessment, Beijing, China

**Keywords:** *Vibrio vulnificus*, genome-wide association studies (GWAS), genome-wide epistasis studies (GWES), potential pathogenic targets, survival strategies

## Abstract

*Vibrio vulnificus*, a foodborne pathogen, has a high mortality rate. Despite its relevance to public health, the identification of virulence genes associated with the pathogenicity of currently known clinical isolates of *V. vulnificus* is incomplete and its synergistic pathogenesis remains unclear. Here, we integrate whole genome sequencing (WGS), genome-wide association studies (GWAS), and genome-wide epistasis studies (GWES), along with phenotype characterization to investigate the pathogenesis and survival strategies of *V. vulnificus*. GWAS and GWES identified a total of six genes (*purH*, *gmr*, *yiaV*, *dsbD*, *ramA*, and *wbpA*) associated with the pathogenicity of clinical isolates related to nucleotide/amino acid transport and metabolism, cell membrane biogenesis, signal transduction mechanisms, and protein turnover. Of these, five were newly discovered potential specific virulence genes of *V. vulnificus* in this study. Furthermore, GWES combined with phenotype experiments indicated that *V. vulnificus* isolates were clustered into two ecological groups (EGs) that shared distinct biotic and abiotic factors, and ecological strategies. Our study reveals pathogenic mechanisms and their evolution in *V. vulnificus* to provide a solid foundation for designing new vaccines and therapeutic targets.

## 1 Introduction

*Vibrio vulnificus* is an opportunistic Gram-negative pathogenic bacterium broadly distributed in estuarine and coastal waters that typically infects people through consumption of tainted raw seafood or direct contact with seawater through open wounds. Primary septicemia is the most lethal consequence of *V. vulnificus* foodborne infection, with a mortality rate of more than 50% in immunocompromised patients, while wound infection has a mortality rate of 17% (Jones and Oliver, 2009). The distribution of *V. vulnificus* is related to temperature. With global climate change and the increase in seawater temperature, the number of infection cases and the geographical distribution of the pathogen are expanding (Motes et al., 1998).

Given its potential as an emerging infectious disease, an understanding of the variation in and distribution of *V. vulnificus* strains is of high priority. Molecular typing methods used to date include the polymerase chain reaction (PCR) analysis of variations in the virulence-correlated gene (*vcg*) (Warner and Oliver, 2008), 16S rRNA gene sequence analysis (Nilsson et al., 2003), multi-locus enzyme electrophoresis (MLEE), random amplification of polymorphic DNA (RAPD) (Gutacker et al., 2003), multi-locus sequence typing (MLST) (Bisharat et al., 2005; Sanjuán et al., 2011), and the genome-wide core-single nucleotide polymorphism (SNP) phylogenetic tree (Roig et al., 2018). Traditional typing methods have classified *V. vulnificus* into three biotypes. However, the genome-wide core-SNP phylogenetic tree provides better resolution for reconstructing relationships among samples than traditional methods, has classified *V. vulnificus* isolates fall into 5 lineages (Roig et al., 2018). However, currently published data on the phylogenetic structure of *V. vulnificus* have a small sample size and sampling bias (e.g., no Chinese mainland isolates are included). Thus, expanding the *V. vulnificus* genome dataset is necessary for a comprehensive reconstruction of the phylogenetic structure of the species.

While some virulence factors have been shown to be crucial for *V. vulnificus* pathogenicity (Jones and Oliver, 2009; Pettis and Mukerji, 2020; Yuan et al., 2020), the specific virulence genes of clinical strains of *V. vulnificus* remain unknown. Genome-wide association studies (GWAS) aim to capture associations of genotypes with phenotypes by testing hundreds of thousands of genetic variants across many genomes. GWAS have been successfully used in bacterial research to uncover antibiotic resistance, virulence, host specificity, and prognosis and can potentially be applied to any heritable bacterial traits (Falush and Bowden, 2006). However, bacterial GWAS are also challenging. For example, factors such as the clonal population structure caused by the mode of division and reproduction of bacteria, the vast differences in recombination rates between species, and the high frequency of gene deletions can make it difficult to identify specific variants responsible for phenotypes. In addition, phenotypes are complex traits that are not determined by monogenetic features but rather by the functional interactions of larger groups of gene products. Therefore, it is essential to explore both full sets of causal genetic variants and the complex interactions between genes (epistasis) to increase our understanding of bacterial pathophysiology.

The abundant genome-wide linkage disequilibrium patterns in bacterial genomes make the identification of causal variants problematic. A thorough study of these patterns of genetic variation in populations thus makes it possible to locate complex disease gene complexes, explore the phenotypic diversity of populations, and acquire new insights into the evolutionary history of the species. To date, few studies have analyzed hypothesis-free co-selection of gene variants, known as genome-wide epistasis studies (GWES), to detect co-selection signals in a group (Skwark et al., 2017; Schubert et al., 2019; Cui et al., 2020; Zeng et al., 2020; Chewapreecha et al., 2022), and no data exist for *V. vulnificus*.

In this study, we first analyze the whole genome sequences of 518 V*. vulnificus* isolates and their isolation sources by GWAS. Then, we systematically examine co-adaptation patterns among *V. vulnificus* genetic variants, including core and accessory genome variants using GWES. We identify six genes linked to the pathogenicity of *V. vulnificus* clinical isolates, of which five were newly discovered in this study. We also uncover complicated gene interactions indicating that core and accessory genomes have co-evolved to produce coadapted gene complexes that encode distinct ecological strategies. Our findings indicate that these isolates and the genetic variants encoding them can be characterized by hierarchical clustering into groups that reflect patterns in the evolution of *V. vulnificus*. Finally, we explore the conditions that likely shaped *V. vulnificus* evolution by characterizing the phenotypes of isolates under different environmental conditions. Our results suggest that *V. vulnificus* has gradually altered its fitness landscape through co-adaptation.

## 2 Materials and methods

### 2.1 Bacterial isolates

In total, 518 isolates (from 14 countries, 1964 to 2018) were used in this study, including 325 newly sequenced isolates (29 clinical and 296 environmental) and 193 publicly available isolates (58 clinical, 124 environmental, and 11 unspecified). Detailed sampling information for the *V. vulnificus* isolates used in this study is listed in **Supplementary Table S1**. The genomes of the publicly available isolates were downloaded from the NCBI database (https://www.ncbi.nlm.nih.gov/genome/browse#!/prokaryotes/189/).

### 2.2 Culture conditions

All isolates were taken from the freezer (-80°C), streaked on Columbia blood agar (CBA) plates, and grown at 37°C for 24 hours. A single colony of a strain was transferred to 5 mL Luria-Bertani (LB) medium containing 2% NaCl and cultured at 37°C to the exponential phase with shaking.

### 2.3 DNA preparation

DNA was extracted from samples using the QIAGEN UltraClean® Microbial DNA Isolation kit, Catalog no. 12224-50, as per the manufacturer’s instructions.

### 2.4 Whole-genome sequencing and assembly

Sequencing was performed on the Illumina MiSeq platform with 150 bp paired-end read length. Raw reads with low quality were trimmed with the FASTQ Quality Filter (FASTX-Toolkit) (Pearson et al., 1997). *De novo* assembly of the filtered reads was performed using Shovill version 1.0.4 (Bankevich et al., 2012) with standard parameters. Sequenced isolates had an average genome size of 5.03 Mb and GC content of 46.71%.

### 2.5 Variation detection, phylogenomic analysis, and annotation

SNPs identified from aligning the assemblies to the reference genome YJ016 with the NUCmer module in the software MUMmer 3.0 (Kurtz et al., 2004) were used to describe genetic relationships between isolates. SNPs located in repeated regions with low sequence quality (quality score <20 or covered by <10 reads) were excluded to identify SNPs in the core genome (regions presented in all isolates). After filtering, core-genome SNPs were then used in the maximum likelihood tree (MLTree) construction by RaxML (Stamatakis, 2006), and the MLTree was visualized with iTOL (Letunic and Bork, 2016).

All genomic sequences were annotated using Prokka (Seemann, 2014). We used GFF3 files generated by Prokka passed to Roary (Page et al., 2015) to create a pangenome and output gene presence/absence for each isolate. To obtain additional annotation, we used the pan-gene protein sequences of Roary to BLAST (BLASTP) against the COG and KEGG databases.

### 2.6 Genome-wide association studies

The 518 V*. vulnificus* isolates, including 87 clinical and 420 environmental isolates, were analyzed using the software Pyseer (Lees et al., 2018) and DBGWAS (Jaillard et al., 2018), using the isolate source (clinical or environmental) of *V. vulnificus* as a phenotype.

In Pyseer, the effects of SNPs, insertion and deletion of accessory genes, or *k*-mers on phenotype was evaluated, and the corresponding *P*-value calculated. SNP-based GWAS captures variants in the bacterial core genome. Insertion and deletion of accessory genes-based GWAS captures variants in the bacterial accessory genes insertions and deletions. Finally, *k*-mers are DNA sub-sequences of length *k* (typically 3-100 base pairs), and *k*-mers-based GWAS can reflect diverse genetic events, including SNPs, insertion and deletion of accessory genes, and cover the noncoding regions, including those related to transcriptional and translational regulation, overcoming limitations of analysis at the level of SNPs and non-core gene insertions/deletions (Jaillard et al., 2018).

DBGWAS is a *k*-mer-based GWAS approach that generates interpretable genetic variants linked to diverse phenotypes. Utilizing compacted De Bruijn graphs (cDBG), this method groups cDBG nodes pinpointed by the association model into subgraphs defined by their neighborhood in the initial cDBG. DBGWAS does not require prior annotation or reference genomes. Importantly, it is also computationally efficient.

### 2.7 Genome-wide epistasis and co-selection studies (GWES)

Co-selection analysis was performed separately on the sequence alignment of 518 V*. vulnificus* isolates using the GWES tool SpydrPick (Pensar et al., 2019). SpydrPick hinges on mutual information (MI), which is a general measure of the dependence between two variants. Pairwise analysis of variants by SpydrPick can be performed using core-genome SNPs and pan-genome-wide analyses.

### 2.8 Phenotyping

#### 2.8.1 Bacterial isolates and culture conditions

For the phenotype experiments, five isolates were randomly selected from two EGs (H1 and S1 from EG1; S8, S9, and S13 from EG2). Bacteria were cultured as described above (“Culture Conditions”).

#### 2.8.2 Survival assays

Isolates of *V. vulnificus* were incubated in 2% NaCl-LB medium at 37°C to reach an *A*_600_ (absorbance) of 0.8. We estimated the growth of each culture every hour for 24 hours at different temperatures (4°C, 37°C, and 45°C), different osmotic conditions (2%, 4%, 6%, and 8% NaCl), and varied pH levels (pH 4, pH 5, pH 6, pH 7, pH 8, and pH 9) using *A*_600_. All experiments were carried out in triplicate and repeated three times.

#### 2.8.3 Motility assay

Isolates were inoculated on a swimming plate (LB media containing 0.3% agar) and a swarming plate (LB agar with 3% NaCl) after being cultivated at 37°C overnight to an *A*_600_ of 0.8. Swimming ability was recorded by measuring the diameter of the colony at 12, 24, 36, 48, and 72 h after inoculation onto a swimming plate at 37°C. Swarming ability was recorded after 72 h on a swarming plate at 37°C.

#### 2.8.4 Biofilm formation

The overnight bacterium liquid was added to a 24-well cell culture plate at 37°C for 24 h without shaking; 2% NaCl-LB medium served as a negative control. Each well was rinsed three times with 1× phosphate-buffered saline (PBS) after the supernatant was removed. Adherent bacteria were fixed with methanol for 15 min and then methane was aspirated from the culture wells, leaving them to air dry naturally. Plate cells were then stained with 0.1% crystal violet for 5 min, followed by washing with 1× PBS three times. The bound dye was dissolved with 33% glacial acetic acid. Finally, the optical density of the solution was measured using a Multiskan Spectrum at 590 nm (Narisawa et al., 2005; Yao et al., 2012; Naparstek et al., 2014; Tango et al., 2018; Fan et al., 2020). The experiments were conducted in triplicate, with three repetitions. Biofilm formation was classified as highly positive (*A*_590_ >0.06), low-grade positive (0.03< *A*_590_ ≤0.06), or negative (*A*_590_ ≤0.03).

#### 2.8.5 Anaerobic culture

An anaerobic chamber (Gene Science) filled with a gas mixture comprised of 90% N_2_, 5% CO_2_, and 5% H_2_ was used to create anaerobic conditions. The isolates were inoculated on a Columbia blood agar plate and cultivated at 37°C for 5 days under anaerobic conditions.

#### 2.8.6 Biochemical and antimicrobial susceptibility tests

The VITEK-2 automated microbial identification and drug sensitivity analyzer (BioMérieux) was employed for measuring biochemical parameters and antimicrobial susceptibility.

## 3 Results

### 3.1 Genetic diversity of *Vibrio vulnificus* isolates

We performed whole genome variation detection of 518 *V. vulnificus* isolates from 14 countries between 1964 and 2018. Isolates included 193 isolates from the NCBI database – 58 clinical, 124 environmental, and 11 unspecified; and 325 isolates from newly collected samples – 29 clinical and 296 environmental (**Supplementary Table S1**). From the analysis, 373,704 core-genome SNPs were identified, including 37,386 intergenic SNPs, 211,731 synonymous SNPs, 121,162 nonsynonymous SNPs, and 3,425 nonsense SNPs. The median pairwise SNP distance between all 518 isolates was 24,403, implying a remarkably high genetic diversity among *V. vulnificus* isolates.

**Figure 1:**
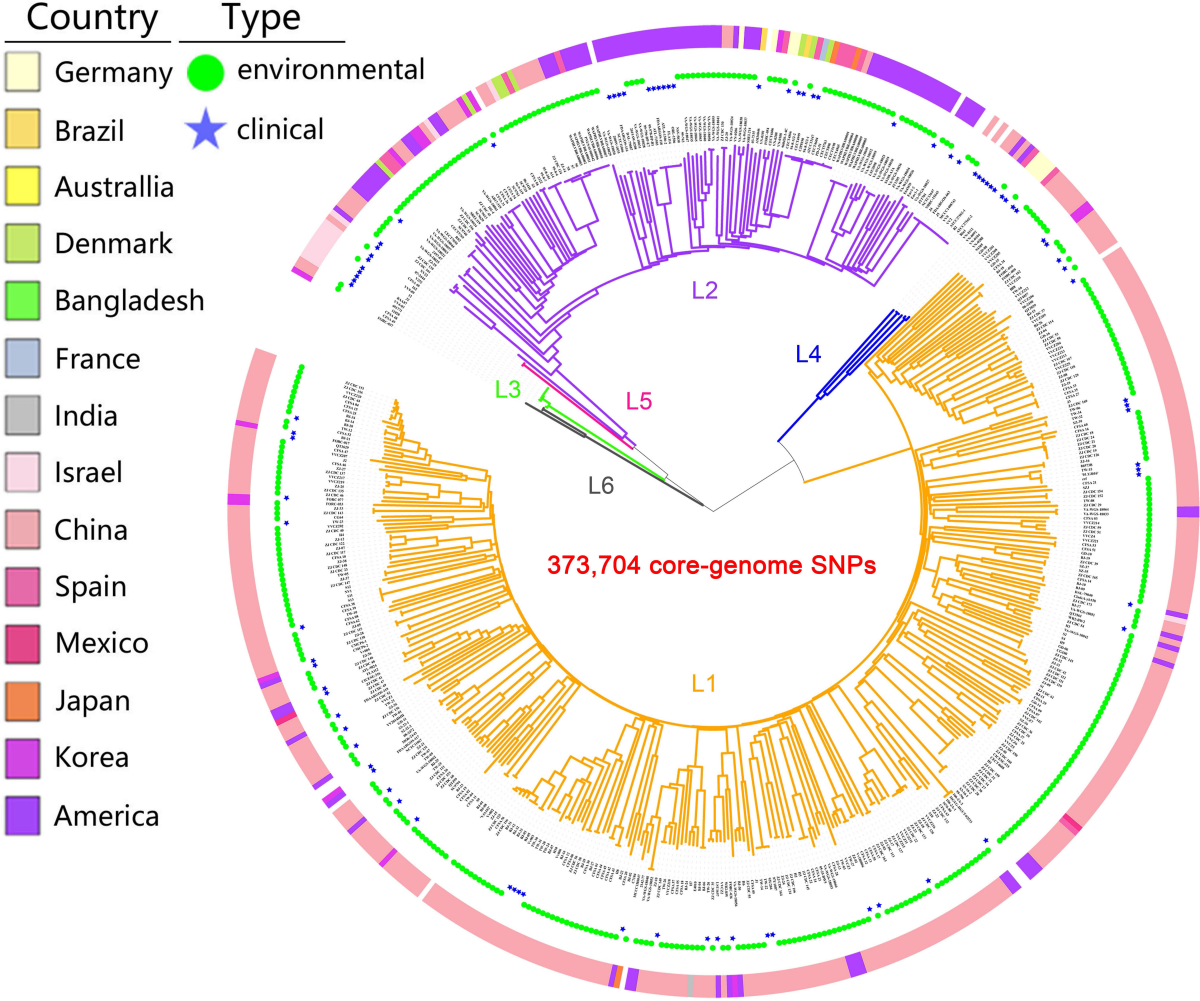
Population structure of *V. vulnificus*. A Maximum Likelihood (ML) tree of 518 V*. vulnificus* isolates was constructed based on 373,704 core-genome SNPs. The ring colors from inner to outer indicate sample type and country of sampling, respectively. Branch colors indicate lineage: orange for L1, purple for L2, green for L3, blue for L4, pink for L5, gray for L6.

A maximum likelihood (ML) tree of the 518 isolates based on core-genome SNPs with *V. vulnificus* YJ016 as the reference sequence divided the species into six well-defined lineages (**Figure 1**). The isolates were mostly in lineages L1 and L2 (93.1%). L1 primarily contained isolates from China (86.8%), India, Mexico, and Bangladesh (100%), with environmental isolates accounting for a large proportion (85.9%). L2 primarily contained isolates from America (50.7%), Denmark, Australia, and France (100%), with environmental isolates accounting for a large proportion (75.4%). L3 only contained biotype 3 isolates from Israel. The number of L3, L4, and L5 isolates was small, but these lineages had a high proportion of clinical isolates (75%). L6 only contained three isolates, of which two were environmental isolates and one was unknown. The overlap in the geographic distributions of the six evolutionary lineages, for both environmental and clinical isolates was considerable, indicating lineages are not type- or geographic location-specific.

### 3.2 Novel pathogenicity-associated variants captured through GWAS

**Figure 2:**
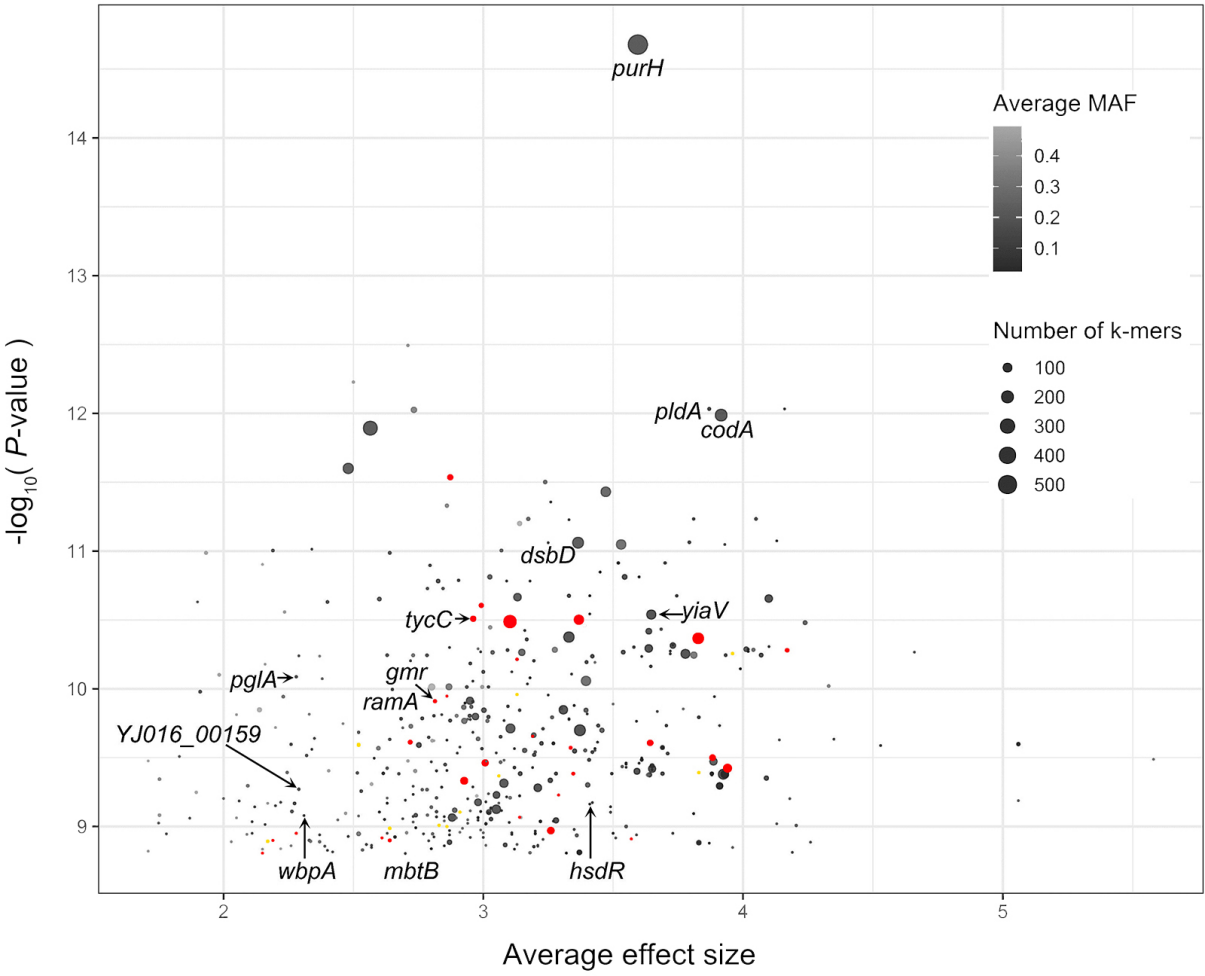
Pyseer results for *V. vulnificus*. The X-axis is average effect size, and Y-axis is the –log_10_(*P*-value) score of Pyseer. Labeled black dots identify genes that carried significant *k*-mers detected in current study. Labeled red dots identify genes that carried both significant *k*-mers and SNPs detected in current study. Labeled yellow dots identify genes that carried both significant *k*-mers and SNPs detected in current study.

To explore novel genetic variants of *V. vulnificus*, we analyzed GWAS results of the 518 *V. vulnificus* isolates. We used Pyseer to identify variants in the whole genome sequences of *V. vulnificus* associated with clinical and environmental phenotypes based on SNPs, insertion and deletion of accessory genes, and *k*-mers, respectively. To reduce the false positive rate, we used a strict threshold to judge the analysis results in combination with prior knowledge (Lees et al., 2018), i.e., only genetic variation with a statistical test *P*-value below 1.74 x 10^-9^ was included [^-^log_10_(*P*-value) >8.76; **Figure 2**]. We discovered 567 genetic variants related to the clinical phenotypes of *V. vulnificus*, located on 567 coding genes at the *k*-mers level. Twenty-eight genetic variants were identified at both the SNPs level and *k*-mers level, and eleven genetic variants were identified at both the insertion and deletion of accessory genes level and *k*-mers level. These variants involved genes that encode structural proteins, transport proteins, metabolic proteins, and signal proteins; thus, they can directly or indirectly affect the pathogenicity of *V. vulnificus* through their roles in energy production and conversion, cell cycle control, translation, ribosomal structure and biogenesis, replication, recombination, and repair, cell wall/membrane/envelope biogenesis, cell motility, intracellular transport, nucleotide/amino acid/carbohydrate/coenzyme/lipid transport and metabolism, inorganic ion transport and metabolism, post-translational modification, protein turnover, chaperone functions, and signal transduction mechanisms. Moreover, the *purH* (encoding bifunctional purine biosynthesis protein PurH) (Kim et al., 2003), and *pldA* (encoding phospholipase A1) (Koo et al., 2007) genes identified in this analysis have previously been experimentally verified as associated with the pathogenicity of *V. vulnificus*, thus confirming the effectiveness of our method.

**Figure 3:**
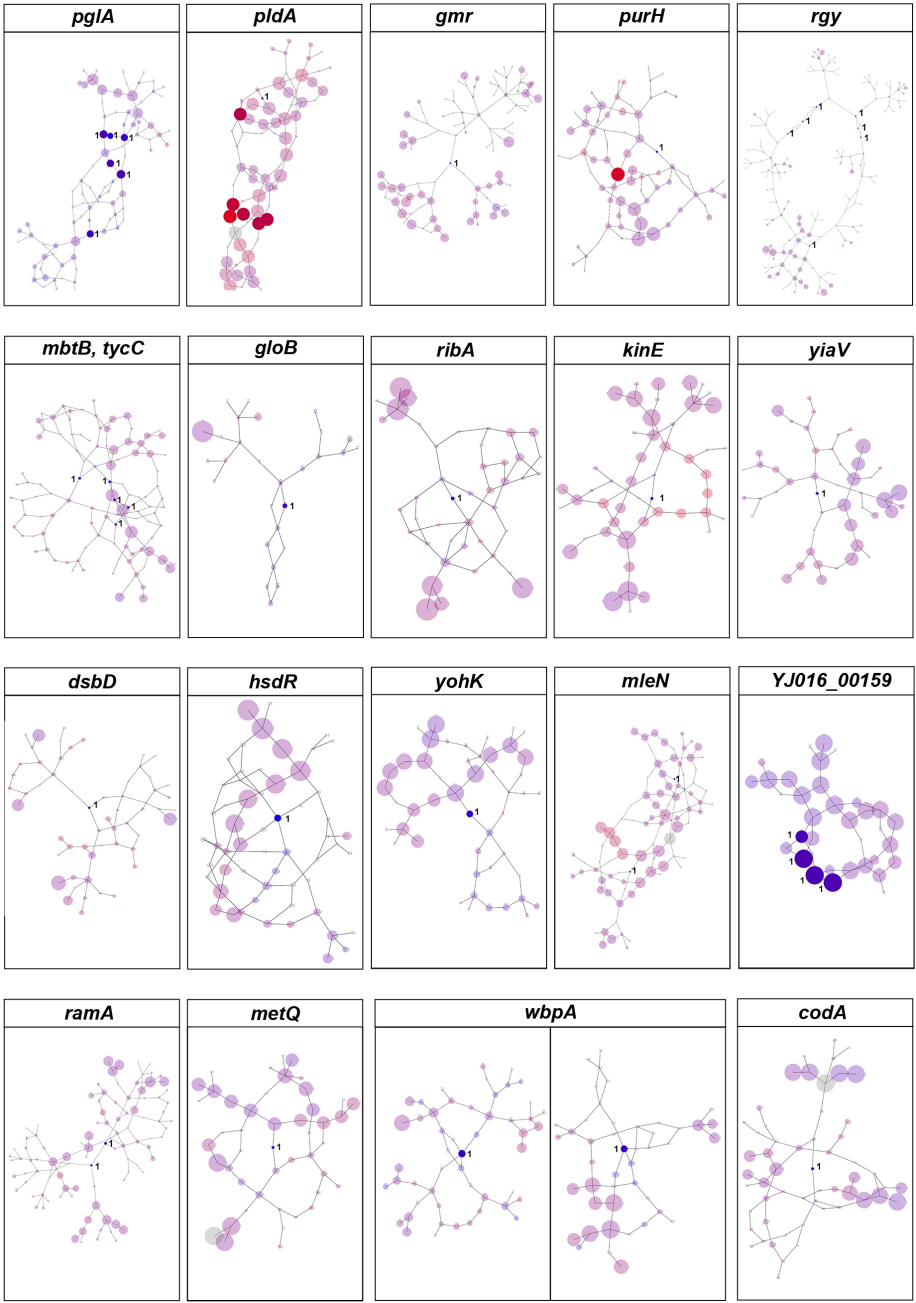
DBGWAS results for *V. vulnificus*. Each subgraph represents a unique genetic event and its mapped genes. Colors are continuously interpolated between blue for clinical phenotype and red for environmental phenotype. Gray indicates untested unitigs (present in > 99% or < 1% of the strains). Insignificant nodes are represented with a degree of transparency. The size of a node is proportional to its allele frequency: the larger the node, the higher the allele frequency.

We also used DBGWAS to test the association between *k*-mers and clinical vs. environmental phenotypes. Overall, we found 100 nodes related to the clinical phenotypes of *V. vulnificus*, located on 20 coding genes [*P*-value = 2.10 x 10^-7^, -log_10_(*P*-value) >6.68; **Figure 3**]. These variants also involved genes that encode structural proteins, transport proteins, metabolic proteins, and signal proteins. The gene products in this group are involved in energy production and conversion, cell membrane biogenesis, nucleotide/amino acid transport and metabolism, protein turnover, and signal transduction mechanisms. In addition, *purH* (Kim et al., 2003), and *pldA* (Koo et al., 2007) genes identified by Pyseer in this analysis were also identified by DBGWAS, which have previously been experimentally verified as associated with the pathogenicity of *V. vulnificus*, thus confirming the effectiveness of our method.

Based on Pyseer and DBGWAS, the clinical virulence or pathogenicity of *V. vulnificus* isolates is associated with thirteen genes, of which eleven (*pglA*, *gmr*, *mbtB*, *tycC*, *yiaV*, *dsbD*, *hsdR*, *YJ016_00159*, *ramA*, *wbpA*, and *codA*) were newly discovered in this study (**Supplementary Table S2**).

### 3.3 Detection of co-selection signals

Current GWAS methods are not accurate enough to identify causal variants, due to extensive genome-wide linkage disequilibrium and they ignore the effect of mutations on protein function (Chen and Shapiro, 2015). Therefore, we performed GWES to identify mutations throughout the genome that are likely to have co-evolved, i.e., potential epistatic interactions between genes.

We used SpydrPick on the whole genome sequences of 518 *V. vulnificus* isolates to assess potential epistatic interactions between genes. Most strong associations occurred on the chromosome between sites within 3 kb. We thus eliminated all sets of associations that spanned less than 3 kb, including those between core/accessory genome elements, to remove associations caused solely by physical linkage, which mask the co-adaptive signals we seek. After screening (extreme outlier threshold ≥0.28), 28,508 co-adaptation groups were identified that could be further subdivided into 166 co-adaptation networks containing 464 core-genome SNPs (53 intergenic SNPs, 221 synonymous SNPs, 177 nonsynonymous SNPs, and 13 nonsense SNPs) on 34 core genes and 750 accessory genes (**Supplementary Table S3**; **Supplementary Figure 1**). The genes included in each co-adaptation group, as well as the corresponding annotation information, are available in **Supplementary Table S4**.

**Figure 4:**
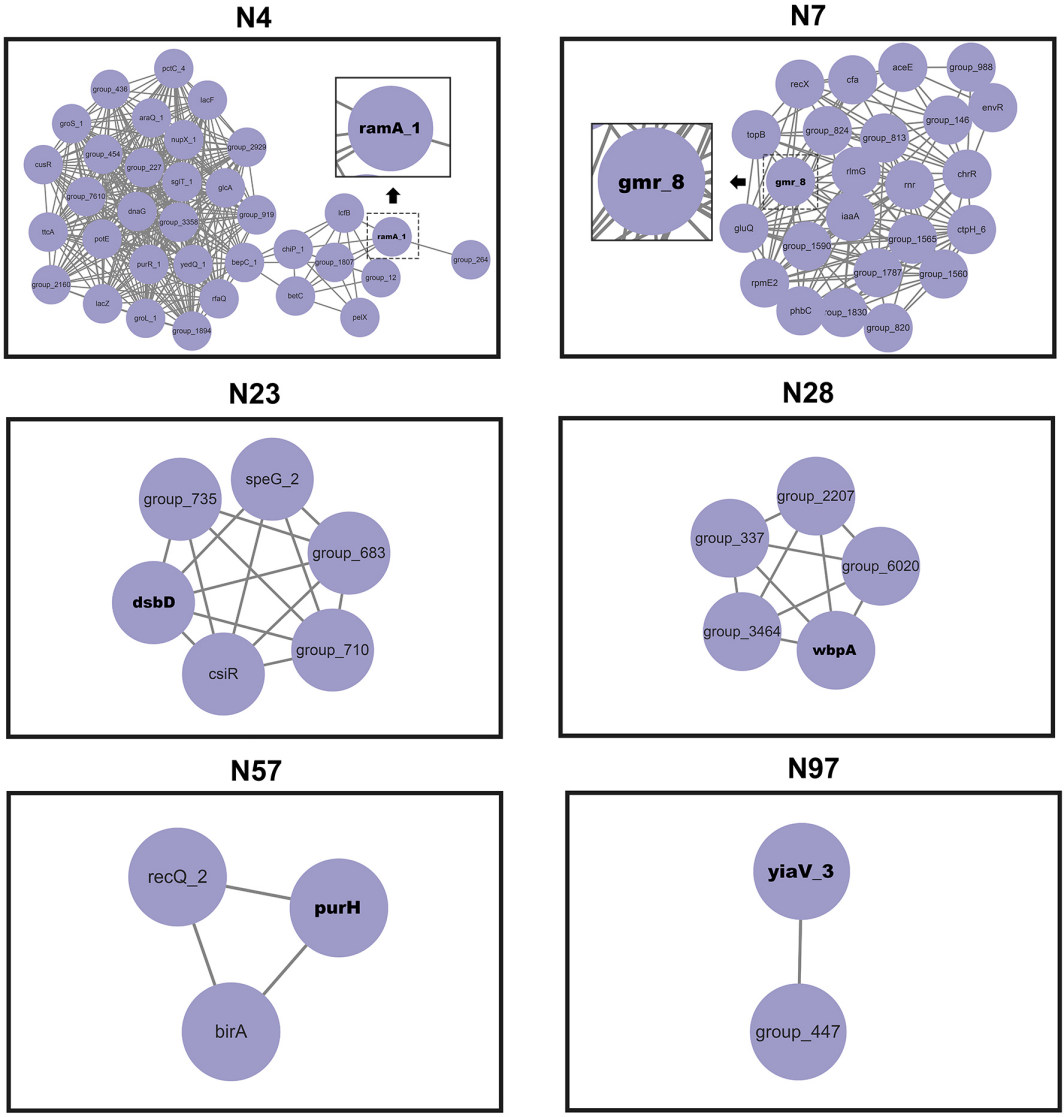
Networks of interacting co-selected gen-gene pairs. Each node denotes a gene under co-selection.

The network of epistatic interactions was examined to identify potential *V. vulnificus* virulence genes. The high-frequency genes in the largest co-adaptation network (N1) were *flgK*, *flgE*, and *flgL* (**Supplementary Table S4**). As a result, we considered *flgK*, *flgL*, and *flgE* genes to be relevant prospective targets of co-selection, i.e., potential *V. vulnificus* virulence genes. The genes *flgK* (Kim et al., 2008), *flgL* (Kim et al., 2008), and *flgE* (Lee et al., 2004) identified in our co-adaptation network have previously been shown (through deletion mutants) to affect the lethality of *V. vulnificus* in mice, lose the ability to form flagella, lose mobility, and exhibit serious defects in cell adhesion and biofilm formation, thus confirming the effectiveness of our method. Our GWES results combined with GWAS results showed that a total of 6 genes, namely *purH*, *gmr*, *yiaV*, *dsbD*, *ramA*, and *wbpA*, were associated with the pathogenicity of *V. vulnificus* clinical isolates (**Figure 4**). The *purH* gene encodes PurH, which is involved in purine nucleotide biosynthesis and catalyzes the last two steps in the *de novo* biosynthesis of IMP (the first nucleotide in the *de novo* purine biosynthesis pathway). The *purH* gene has previously been shown (by deletion mutants) to alter the lethality and cytotoxic activity of *V. vulnificus* in mice and is a virulence gene of *V. vulnificus* (Kim et al., 2003). Five genes, namely *purH*, *gmr*, *yiaV*, *dsbD*, *ramA*, and *wbpA*, were newly discovered in this study. The gene *gmr* (encodes Cyclic di-GMP phosphodiesterase Gmr) regulates the enzyme for synthesis of cyclic di-GMP. Cyclic di-GMP has emerged as one of the most common and essential bacterial second messengers, with the ability to influence biofilm formation, motility, virulence, the cell cycle, differentiation, and other processes, hence influencing *V. vulnificus* pathogenicity (Römling and Amikam, 2006; Römling et al., 2013; Jenal et al., 2017). The gene *yiaV* (encodes inner membrane protein yiaV precursor) (Hu and Coates, 2005; Shimada et al., 2022) and *dsbD* (encodes disulfide interchange protein dsbD precursor) affect antimicrobial drug tolerance of *Escherichia coli (* Missiakas et al., 1995). The gene *ramA* (encodes R-stereoselective amidase) affects antimicrobial drug tolerance of *Klebsiella pneumoniae* (Veleba and Schneiders, 2012). The gene *wbpA* encodes UDP-N-acetyl-D-glucosamine 6-dehydrogenase, which is involved in the synthesis of LPS. It has been previously demonstrated that the *wbpA* deletion mutant can affect the synthesis of LPS in *Pseudomonas aeruginosa*. LPS can evade host defenses, resist phagocytosis and serum-mediated killing and is also a crucial virulence factor for *V. vulnificus* (Burrows et al., 2000; Jones and Oliver, 2009).

### 3.4 Complex structure and ecological group differentiation of co-adaptation networks

**Figure 5:**
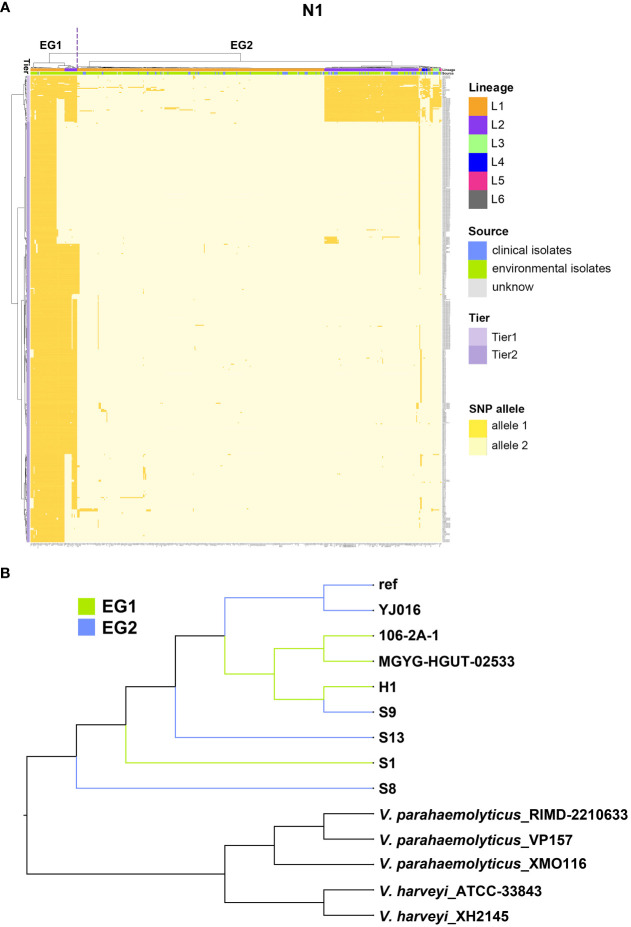
The largest co-adaptation network (N1) identified by SpydrPick. **(A)** The x-axis is the 518 *V. vulnificus* isolates and the right y-axis indicates coadaptation loci. Colors of the heatmap indicate the status of genetic variants, with light yellow/orange for allele1 and allele2 of the SNP allele. The left clustering tree indicates different tiers (Tier 1 and Tier 2). The upper clustering tree indicates (from top to bottom) different EGs (EG1, and EG2), lineages, and sources (clinical vs environmental sample). **(B)** A NJ tree of *Vibrio* isolates. EG1 isolates are indicated with green branches and EG2 isolates with blue branches.

The largest co-adaptation network (N1) included most of the interacting SNPs, which challenged interpretation due to its abundance of pairwise interactions. We thus used hierarchical clustering based on N1 variants to classify variants into two ecological groups (EGs), EG1 and EG2 (**Figure 5A**). EG1 contained L1 and L2 variants, while EG2 included variants within L1, L2, L3, L4, L5, and L6. EG1 isolates (1/59) had a lower percentage of clinical samples than EG2 isolates (86/459), indicating a lower virulence potential of this ecological group in humans. Despite the substantial number of coadaptation-related differences between EG1 and EG2, both ecological groups had no obstacle to gene exchange between groups in most regions of the genome (**Figure 5B**).

Given the complex structure of the co-adaptation network N1, we also did a cluster analysis of the coadaptation loci, which separated the coadapted complex loci into two tiers. Tier 1 loci variants were stable in EG1 but highly variable in EG2, containing genes *csgD*, *glmM*, *glmU*, *mrdB*, *flgK*, and *flgL*. Tier 2 loci variants broadly distinguished the two EGs, containing most of the significant coadapted loci (genes *glmU*, *mrdB*, *flgK*, *flgL*, *flgE*, *spovD*, *alr*, *clpB*, and *glxR*) identified by SpydrPick.

We detected 4 co-adaptation networks (N54, N78, N79, and N80) involving SNPs that were interactions of the type “incompatibility”, meaning that when one or more genes exist, at least other genes are absent. We detected a further 161 co-adaptation networks (N2-N53, N55-N77, and N81-N166) involving accessory genes that were “genome island-like”.

### 3.5 Multidimensional phenotyping

**Figure 6:**
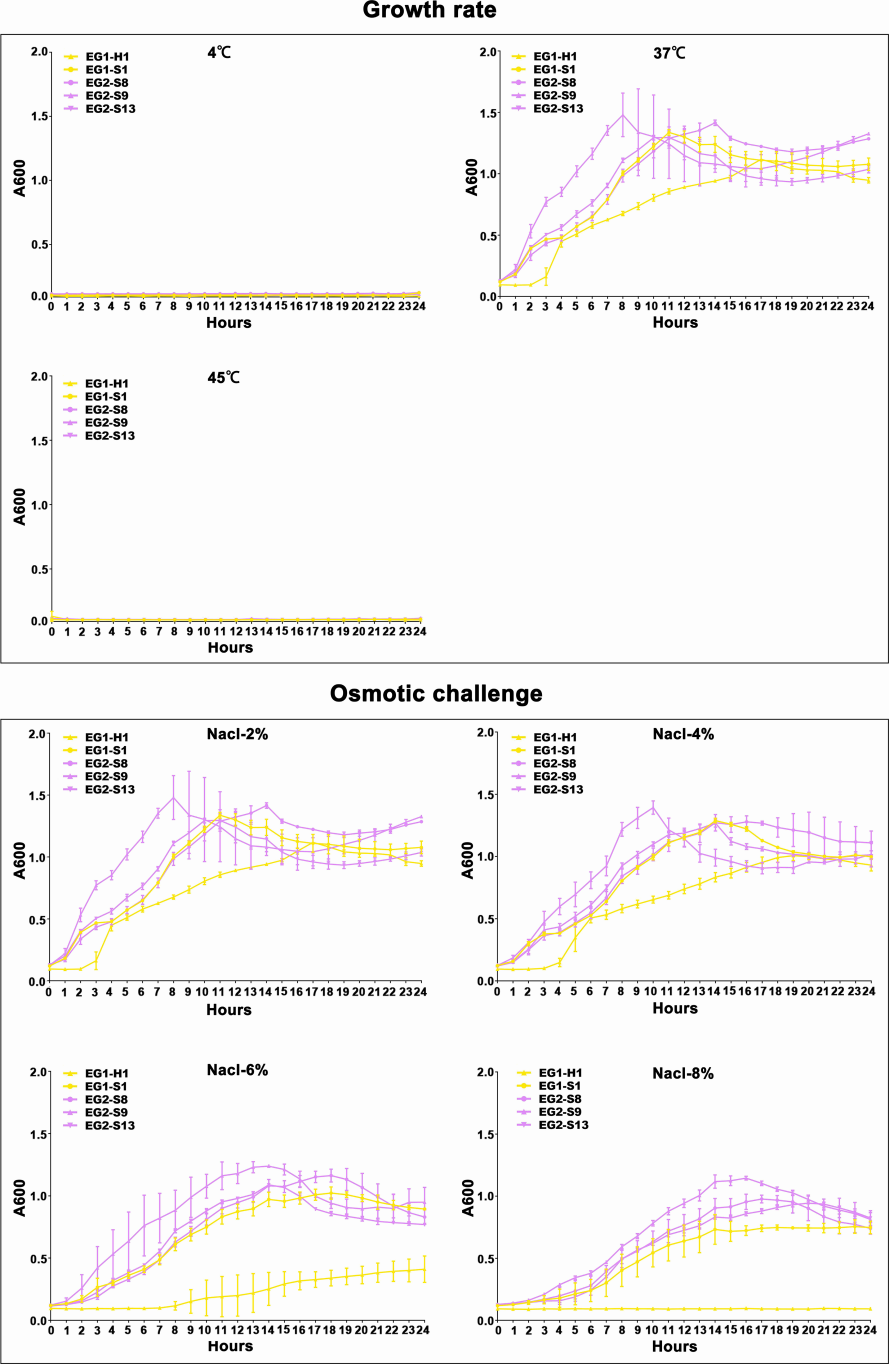
Temperature and salinity survival assays of *V. vulnificus* isolates from two EGs. Growth curves of isolates from two EGs measured using A_600_ at different temperatures (4°C, 37°C, and 45°C), and salinities (2% NaCl, 4% NaCl, 6% NaCl, 8% NaCl).

**Figure 7:**
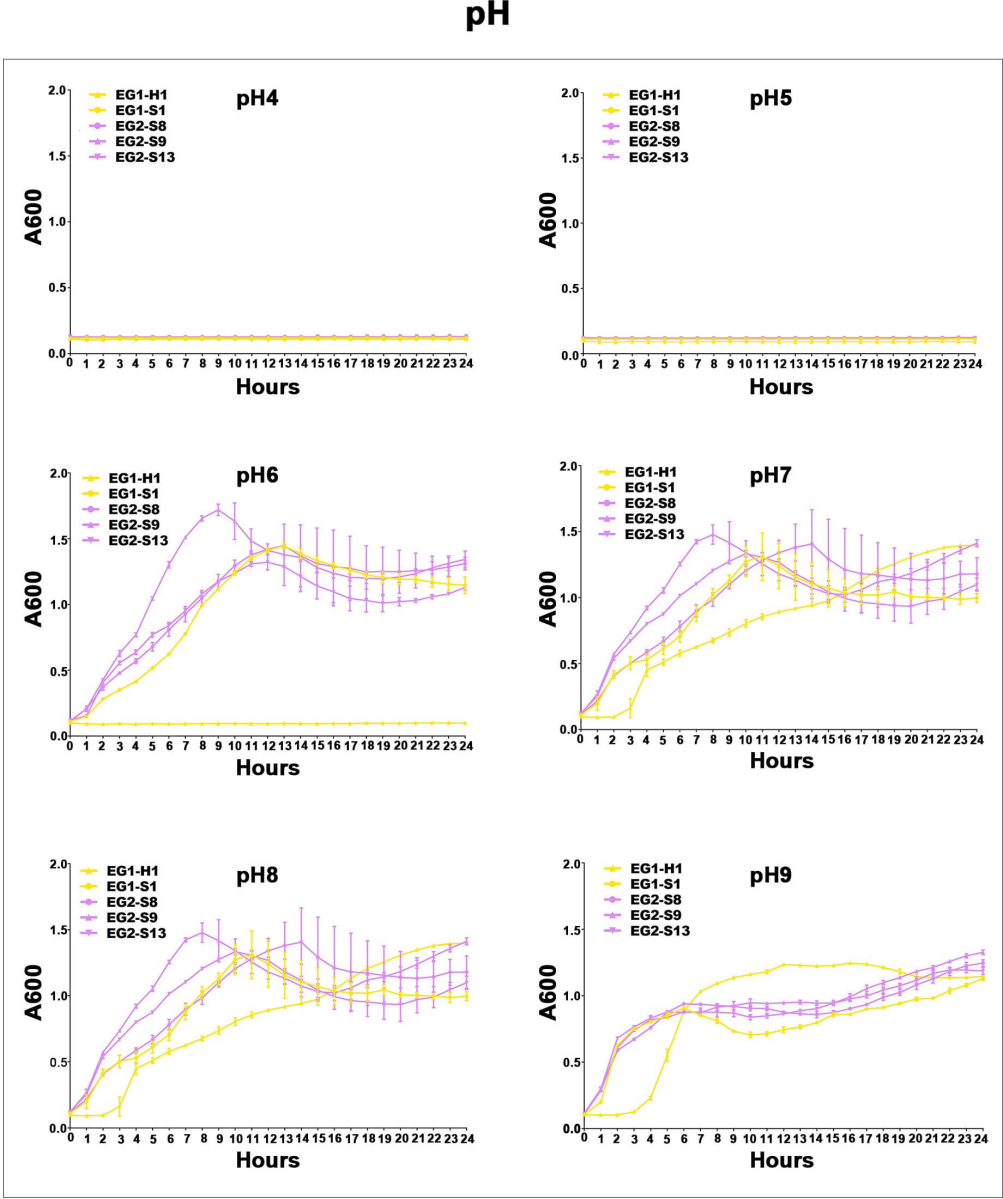
Acid-base survival assays of *V. vulnificus* isolates from two EGs. Growth curves of isolates from two EGs measured using A_600_ at different pH (pH 4, pH 5, pH 6, pH 7, pH 8, pH 9).

**Figure 8:**
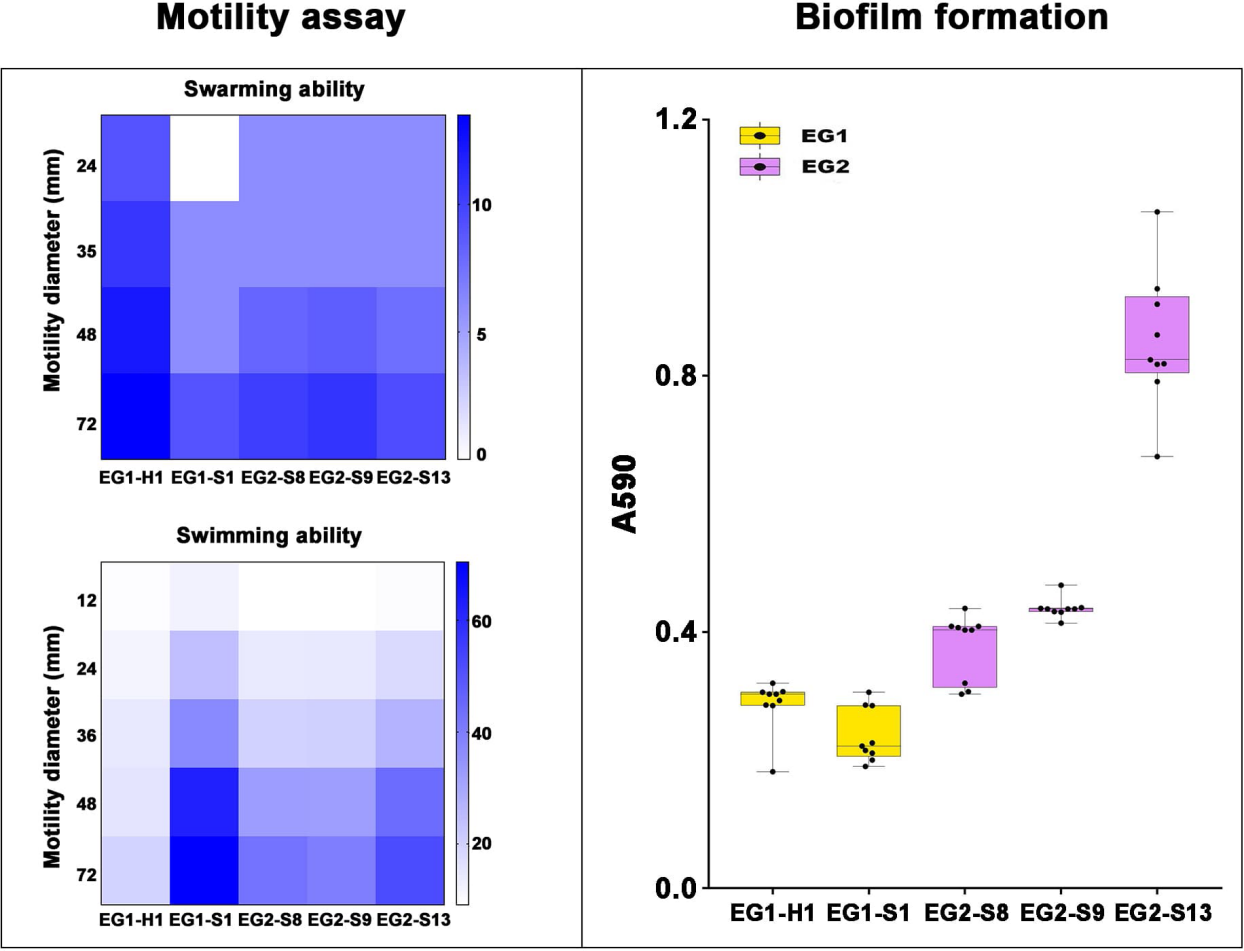
Motility assay and biofilm formation of *V. vulnificus* isolates from two EGs. Motility assay: Swarming (top) and Swimming (bottom) ability of isolates from two EGs were measured by motility diameters in swimming and swarming plates. Biofilm formation: Biofilm formation ability was measured using A_590_.

To assess the potential role of coadapted gene complexes on ecologically important phenotypic traits, we conducted a preliminary investigation of isolates from the two EGs (n = 5, 2 isolates for EG1, 3 isolates for EG2) to measure relevant phenotypes: growth rate under temperature challenges (4°C, 37°C, and 45°C), osmotic challenges (2, 4, 6, and 8% NaCl), and pH challenges (pH 4, 5, 6, 7, 8 and 9); motility assay, biofilm formation ability, survival under anaerobic conditions, biochemical parameters, and antimicrobial susceptibility. Neither EG1 nor EG2 isolates grew at 4°C or 45°C. EG2 isolates had a faster growth rate than EG1 isolates at 37°C. All *V. vulnificus* grew best at 2% NaCl concentration, but EG2 isolates were more tolerant to high salt environments than EG1 isolates (**Figure 6**). The growth states of the two ecological groups were similar at acidic (pH 4-5) and alkaline (pH 9) conditions. EG2 isolates grew faster than EG1 isolates at pH 6-8. In addition, acidic conditions (pH 4-5) are not suitable for the growth and reproduction of *V. vulnificus*. Neutral conditions (pH 6-7) favor the growth and reproduction of *V. vulnificus*. Alkaline conditions (pH 8-9) not only impede the growth and reproduction of *V. vulnificus* but also inhibit its activity (**Figure 7**). The growth variation of two ecological groups under temperature challenges, osmotic challenges and pH challenges occurred mainly during the stabilization and senescence phases of bacterial growth, with little change during the logarithmic growth period. In motility assay, both EG1 and EG2 isolates had strong swimming and swarming abilities, but there was no difference (**Figure 8**). The biofilm formation ability of EG2 isolates was stronger than that of EG1 isolates (**Figure 8**). As expected, all isolates failed to grow under strict anaerobic conditions (**Supplementary Figure 2**). Biochemical assays detected no difference among isolates (**Supplementary Table S5**). Minimal inhibitory concentration (MIC) values were employed to assess antimicrobial susceptibility. Isolates were sensitive to most of the antibiotics tested, but insensitive to cefazolin (**Supplementary Table S6**). Cefazolin, as a first-generation cephalosporin, has limited effectiveness against Gram-negative bacteria, making them more susceptible to developing resistance. This is attributed to the relatively weak stability of cefazolin to β-lactamase produced by Gram-negative bacteria. Previous studies have reported the resistance of *V. vulnificus* to cefazolin (Wang et al., 2009; Pan et al., 2013).

## 4 Discussion

Our results combined GWAS and GWES and applied them to the field of *V. vulnificus* for the first time. In this study, we used a robust mix of WGS, GWAS, and GWES, as well as phenotype description, to analyze 518 genomes of *V. vulnificus* isolates collected worldwide from a variety of sources to investigate genetic diversity, virulence genes, the drivers of genotypic and phenotypic evolution, and the emergence of coadapted gene complexes. Our phylogenomic analysis indicates that *V. vulnificus* has diverged into six distinct lineages, L1 through L6. This result improves our resolution of *V. vulnificus* population structure, identifying one more lineage than a previous study (Roig et al., 2018). The six lineages overlap extensively across biogeographic regions. Given that *V. vulnificus* cannot permanently colonize large animals, seabirds, or other aquatic animals that migrate long distances, we speculate that human activities, such as shipping and global trade in aquatic products, have facilitated the spread of *V. vulnificus* across oceans, allowing for genetic exchange between bacteria from different seas. This has ultimately affected the structural composition and evolutionary patterns of bacterial populations.

According to our GWAS results, thirteen genes are associated with the pathogenicity of *V. vulnificus* clinical isolates, of which eleven were newly discovered in this study. The *purH* (Narisawa et al., 2005), and *pldA* (Naparstek et al., 2014) genes identified in our analysis have been experimentally linked with the pathogenicity of *V. vulnificus*. These findings highlight the reliability of our methodology and the potential of GWAS for investigating pathogenesis in bacteria.

We were successful in examining potential epistatic interactions using GWES to identify co-evolved proteins and thus hypothesize potential networks of functionally linked genes. Our GWES results combined with GWAS results showed that a total of six genes correlate with the pathogenicity of *V. vulnificus* clinical isolates, of which five (*gmr*, *yiaV*, *dsbD*, *ramA*, and *wbpA*) were newly discovered in this study. Our findings can be applied to find candidate targets for vaccine development against *V. vulnificus* and provide an important foundation for further insight into the pathogenic mechanism of this species. Future studies should consider gene knockout and *in vivo* infection experiments on these targets.

Furthermore, we discovered that all *V. vulnificus* isolates could be separated into two EGs based on core-genome SNPs by GWES. To further elucidate the evolutionary drivers behind the divergence of the two EGs, we conducted phenotypic experiments. The two EGs exhibit distinct ecologies based on our investigation of *V. vulnificus* phenotypes, genomic evolution, and distribution. EG1 members appear to be more adapted to low-nutrient, lower-salinity, and brackish-like water environments, whereas EG2 members were more likely to come from rich-nutrient, higher-salinity, ocean-like environments. Our phenotypic experiments indicate EG2 isolates can withstand a wider range of stressors and have a competitive advantage in colonizing and growing in diverse hosts, which might explain why most isolates were obtained from this cluster. It was noteworthy that the proportion of clinical samples in EG1 isolates (1/59) was lower than that in EG2 isolates (86/459), indicating a lower virulence potential of this ecological group in humans. Interesting findings here include the clear presence of clinical isolates in both EG1 and EG2 that confirm work that “clinical isolates” are not phylogenetically grouped. We speculate that these phylogenetically distant clusters occupy distinct niches that may differ in their natural hosts or habitats, and this physical isolation may lead to obvious evolutionary pressures that reduce the likelihood of encounters and recombination, leading to genetic isolation and the emergence of distinct ecotypes, with potentially devastating consequences for aquaculture and human health. Despite unresolved issues, our findings emphasize the central role of lateral motility in constructing ecologically significant variation within the species. We also found that the co-adaptation networks went through progressive stages, consistent with the previous research results in *Vibrio parahaemolyticus* (Cui et al., 2020), such as accidental combination, semi-stability, stability, and the emergence of new species. These data provide insight into the evolution of *V. vulnificus* and its potential as an emerging infectious disease.

## 5 Conclusions

We combined WGS, GWAS, and GWES, as well as detailed phenotypic analysis of *V. vulnificus* to reveal the presence of six phylogenetic lineages that are related to the geographical distribution of isolates. Human activities such as shipping and trade in global aquatic products may contribute to the trans-oceanic transmission of *V. vulnificus*. The results obtained from GWAS and GWES reveal complex genomic variations, providing a rigorous prediction of which genes were essential for *V. vulnificus* clinical isolates’ pathogenicity. Particularly, genes *gmr*, *yiaV*, *dsbD*, *ramA*, and *wbpA* were identified as hotspots of the co-selection maps. Additionally, *V. vulnificus* isolates were clustered into two EGs (EG1 and EG2) with distinct patterns of bacterial behavior in our investigation. Our study can be utilized to better understand the pathogenic mechanism and evolution of *V. vulnificus*.

## Data availability statement

The datasets presented in this study can be found in online repositories. The names of the repository/repositories and accession number(s) can be found in the article/**Supplementary Material**.

## Author contributions

J-XZ: Conceptualization, Data curation, Formal Analysis, Investigation, Methodology, Software, Validation, Visualization, Writing - original draft, Writing - review & editing. YY: Conceptualization, Writing - review & editing. Q-HH: Resources, Writing - review & editing. D-ZJ: Resources, Writing - review & editing. YB: Resources, Writing - review & editing. W-WX: Conceptualization, Funding acquisition, Project administration, Resources, Supervision, Writing - review & editing. LK: Conceptualization, Funding acquisition, Project administration, Resources, Supervision, Writing - review & editing. J-LW: Conceptualization, Funding acquisition, Project administration, Resources, Supervision, Writing - review & editing. 

## References

[B2] Bankevich A. Nurk S. Antipov D. Gurevich A. A. Dvorkin M. Kulikov A. S.. (2012). SPAdes: a new genome assembly algorithm and its applications to single-cell sequencing. J. Comput. Biol. 19 (5), 455–477. doi: 10.1089/cmb.2012.0021 22506599 PMC3342519

[B3] Bisharat N. Cohen D. I. Harding R. M. Falush D. Crook D. W. Peto T.. (2005). Hybrid *vibrio vulnificus* . Emerging. Infect. Dis. 11 (1), 30. doi: 10.3201/eid1101.040440 PMC3294331 15705319

[B4] Burrows L. L. Pigeon K. E. Lam J. S. (2000). *Pseudomonas aeruginosa* B-band lipopolysaccharide genes *wbpA* and *wbpI* and their *Escherichia coli* homologues *wecC* and *wecB* are not functionally interchangeable. FEMS Microbiol. Lett. 189 (2), 135–141. doi: 10.1111/j.1574-6968.2000.tb09219.x 10930727

[B5] Chen P. E. Shapiro B. J. (2015). The advent of genome-wide association studies for bacteria. Curr. Opin. Microbiol. 25, 17–24. doi: 10.1016/j.mib.2015.03.002 25835153

[B6] Chewapreecha C. Pensar J. Chattagul S. Pesonen M. Sangphukieo A. Boonklang P.. (2022). Co-evolutionary signals identify Burkholderia pseudomallei survival strategies in a hostile environment. Mol. Biol. Evol. 39 (1), msab306. doi: 10.1093/molbev/msab306 34662416 PMC8760936

[B7] Cui Y. Yang C. Qiu H. Wang H. Yang R.F. Falush D.. (2020). The landscape of coadaptation in *Vibrio parahaemolyticus* . Elife 9, e54136. doi: 10.7554/eLife.54136.sa2 32195663 PMC7101233

[B8] Falush D. Bowden R. (2006). Genome-wide association mapping in bacteria? Trends Microbiol. 14 (8), 353–355. doi: 10.1016/j.tim.2006.06.003 16782339

[B9] Fan Y. Qiao J. Lu Z. Fen Z. Y. Tao Y. Lv F. X.. (2020). Influence of different factors on biofilm formation of *Listeria monocytogenes* and the regulation of *cheY* gene. Food Res. Int. 137, 109405. doi: 10.1016/j.foodres.2020.109405 33233092

[B10] Gutacker M. Conza N. Benagli C. Pedroli A. Bernasconi M. V. Permin L.. (2003). Population genetics of *Vibrio vulnificus*: identification of two divisions and a distinct eel-pathogenic clone. Appl. Environ. Microbiol. 69 (6), 3203–3212. doi: 10.1128/AEM.69.6.3203-3212.2003 12788717 PMC161503

[B11] Hu Y. Coates A. R. M. (2005). Transposon mutagenesis identifies genes which control antimicrobial drug tolerance in stationary-phase *Escherichia coli* . FEMS Microbiol. Lett. 243 (1), 117–124. doi: 10.1016/j.femsle.2004.11.049 15668009

[B12] Jaillard M. Lima L. Tournoud M. Mahé P Belkum A. V. Lacroix V. (2018). A fast and agnostic method for bacterial genome-wide association studies: Bridging the gap between *K*-mers and genetic events. PloS Genet. 14 (11), e1007758. doi: 10.1371/journal.pgen.1007758 30419019 PMC6258240

[B13] Jenal U. Reinders A. Lori C. (2017). Cyclic di-GMP: second messenger extraordinaire. Nat. Rev. Microbiol. 15 (5), 271–284. doi: 10.1038/nrmicro.2016.190 28163311

[B14] Jones M. K. Oliver J. D. (2009). *Vibrio vulnificus*: disease and pathogenesis. Infect. Immun. 77 (5), 1723–1733. doi: 10.1128/IAI.01046-08 19255188 PMC2681776

[B15] Kim S. Y. Hong H. Y. Rhee J. H.. (2008). Roles of flagellar hook-associated proteins in *Vibrio vulnificus* motility and virulence. J. Bacteriol. Virol. 38 (1), 1–10. doi: 10.4167/jbv.2008.38.1.1

[B16] Kim Y. R. Lee S. E. Kim C. M. Kim S. Y. Shin E. K. Shin D. H.. (2003). Characterization and pathogenic significance of *Vibrio vulnificus* antigens preferentially expressed in septicemic patients. Infect. Immun. 71 (10), 5461–5471. doi: 10.1128/IAI.71.10.5461-5471.2003 14500463 PMC201039

[B17] Koo B. S. Lee J. H. Kim S. C. Yoon H. Y. Kim K. A. Kwon K. B.. (2007). Phospholipase A as a potent virulence factor of *Vibrio vulnificus* . Int. J. Mol. Med. 20 (6), 913–918. doi: 10.3892/ijmm.20.6.913 17982702

[B18] Kurtz S. Phillippy A. Delcher A. L. Smoot M. Shumway M. Antonescu C.. (2004). Versatile and open software for comparing large genomes. Genome Biol. 5, 1–9. doi: 10.1186/gb-2004-5-2-r12 PMC395750 14759262

[B19] Lee J. H. Rho J. B. Park K. J. Kim C. B. Han Y. S. Choi S. H.. (2004). Role of flagellum and motility in pathogenesis of *Vibrio vulnificus* . Infect. Immun. 72 (8), 4905–4910. doi: 10.1128/IAI.72.8.4905-4910.2004 15271959 PMC470688

[B20] Lees J. A. Galardini M. Bentley S. D. Veazey J. E. Hunsucker J. C. Garthright W. E.. (2018). Pyseer: a comprehensive tool for microbial pangenome-wide association studies. Bioinformatics 34 (24), 4310–4312. doi: 10.1093/bioinformatics/bty539 30535304 PMC6289128

[B21] Letunic I. Bork P. (2016). Interactive tree of life (iTOL) v3: an online tool for the display and annotation of phylogenetic and other trees. Nucleic Acids Res. 44 (W1), W242–W245. doi: 10.1093/nar/gkw290 27095192 PMC4987883

[B22] Missiakas D. Schwager F. Raina S. (1995). Identification and characterization of a new disulfide isomerase-like protein (DsbD) in *Escherichia coli* . EMBO J. 14 (14), 3415–3424. doi: 10.1002/j.1460-2075.1995.tb07347.x 7628442 PMC394408

[B23] Motes M. L. DePaola A. Cook D. W. Veazey J. E. Hunsucker J. C. Garthright W. E. (1998). Influence of water temperature and salinity on *Vibrio vulnificus* in Northern Gulf and Atlantic Coast oysters (Crassostrea virginica). Appl. Environ. Microbiol. 64 (4), 1459–1465. doi: 10.1128/AEM.64.4.1459-1465.1998 9546182 PMC106170

[B24] Naparstek L. Carmeli Y. Navon-Venezia S. Banin E. (2014). Biofilm formation and susceptibility to gentamicin and colistin of extremely drug-resistant KPC-producing *Klebsiella pneumoniae* . J. Antimicrob. Chemother. 69 (4), 1027–1034. doi: 10.1093/jac/dkt487 24408988

[B25] Narisawa N. Furukawa S. Ogihara H. Yamasaki M. (2005). Estimation of the biofilm formation of *Escherichia coli* K-12 by the cell number. J. Biosci. Bioeng. 99 (1), 78–80. doi: 10.1263/jbb.99.78 16233759

[B26] Nilsson W. B. Paranjype R. N. DePaola A. Strom M. S. (2003). Sequence polymorphism of the 16S rRNA gene of *Vibrio vulnificus* is a possible indicator of strain virulence. J. Clin. Microbiol. 41 (1), 442–446. doi: 10.1128/JCM.41.1.442-446.2003 12517889 PMC149629

[B27] Page A. J. Cummins C. A. Hunt M. Wong V. K. Reuter S. Holden M. T.G.. (2015). Roary: rapid large-scale prokaryote pan genome analysis. Bioinformatics 31 (22), 3691–3369. doi: 10.1093/bioinformatics/btv421 26198102 PMC4817141

[B28] Pan J. Zhang Y. Jin D. Jin D. Z. Ding G. Q. Luo Y.. (2013). Molecular characterization and antibiotic susceptibility of *Vibrio vulnificus* in retail shrimps in Hangzhou, People's Republic of China. J. Food Prot. 76 (12), 2063–2068. doi: 10.4315/0362-028X.JFP-13-161 24290683

[B29] Pearson W. R. Wood T. Zhang Z.. (1997). Comparison of DNA sequences with protein sequences. Genomics 46 (1), 24–36. doi: 10.1006/geno.1997.4995 9403055

[B30] Pensar J. Puranen S. Arnold B. MacAlasdair N. Kuronen J. Tonkin-Hill G.. (2019). Genome-wide epistasis and co-selection study using mutual information. Nucleic Acids Res. 47 (18), e112–e112. doi: 10.1093/nar/gkz656 31361894 PMC6765119

[B31] Pettis G. S. Mukerji A. S. (2020). Structure, function, and regulation of the essential virulence factor capsular polysaccharide of *Vibrio vulnificus* . Int. J. Mol. Sci. 21 (9), 3259. doi: 10.3390/ijms21093259 32380667 PMC7247339

[B32] Roig F. J. González-Candelas F. Sanjuan E. Fouz B. Feil E. J. Llorens C.. (2018). Phylogeny of *Vibrio vulnificus* from the analysis of the core-genome: implications for intra-species taxonomy. Front. Microbiol. 8, 2613. doi: 10.3389/fmicb.2017.02613 29358930 PMC5765525

[B33] Römling U. Amikam D. (2006). Cyclic di-GMP as a second messenger. Curr. Opin. Microbiol. 9 (2), 218–228. doi: 10.1016/j.mib.2006.02.010 16530465

[B34] Römling U. Galperin M. Y. Gomelsky M. (2013). Cyclic di-GMP: the first 25 years of a universal bacterial second messenger. Microbiol. Mol. Biol. Rev. 77 (1), 1–52. doi: 10.1128/mmbr.00043-12 23471616 PMC3591986

[B35] Sanjuán E. González-Candelas F. Amaro C. (2011). Polyphyletic origin of *Vibrio vulnificus* biotype 2 as revealed by sequence-based analysis. Appl. Environ. Microbiol. 77 (2), 688–695. doi: 10.1128/AEM.01263-10 21097581 PMC3020543

[B36] Schubert B. Maddamsetti R. Nyman J. Farhat M. R. Marks D. S. (2019). Genome-wide discovery of epistatic loci affecting antibiotic resistance in *Neisseria gonorrhoeae* using evolutionary couplings. Nat. Microbiol. 4 (2), 328–338. doi: 10.1038/s41564-018-0309-1 30510172 PMC6663919

[B37] Seemann T. (2014). Prokka: rapid prokaryotic genome annotation. Bioinformatics 30 (14), 2068–2069. doi: 10.1093/bioinformatics/btu153 24642063

[B38] Shimada T. Murayama R. Mashima T. Kawano N Ishihama A. (2022). Regulatory role of CsuR (YiaU) in determination of cell surface properties of *Escherichia coli* K-12. Microbiology 168 (4), 001166. doi: 10.1099/mic.0.001166 35438626

[B39] Skwark M. J. Croucher N. J. Puranen S. Chewapreecha C. Pesonen M. Xu Y.Y.. (2017). Interacting networks of resistance, virulence and core machinery genes identified by genome-wide epistasis analysis. PloS Genet. 13 (2), e1006508. doi: 10.1371/journal.pgen.1006508 28207813 PMC5312804

[B40] Stamatakis A. (2006). RAxML-VI-HPC: maximum likelihood-based phylogenetic analyses with thousands of taxa and mixed models. Bioinformatics 22 (21), 2688–2690. doi: 10.1093/bioinformatics/btl446 16928733

[B41] Tango C. N. Akkermans S. Hussain M. S. Khan I. Impe J. J. Jin Y. G.. (2018). Modeling the effect of pH, water activity, and ethanol concentration on biofilm formation of *Staphylococcus aureus* . Food Microbiol. 76, 287–295. doi: 10.1016/j.fm.2018.06.006 30166152

[B42] Veleba M. Schneiders T. (2012). Tigecycline resistance can occur independently of the *ramA* gene in *Klebsiella pneumoniae* . Antimicrob. Agents Chemother. 56 (8), 4466–4467. doi: 10.1128/AAC.06224-11 22644034 PMC3421586

[B43] Wang Z. Shao P. Wu X. Ni K. Lu H. (2009). *Vibrio vulnificus*: cultivation, identification and antimicrobial susceptibility. Chin. J. Clin. Infect. Dis. 19 (6), 293–296.

[B44] Warner E. Oliver J. D. (2008). Population structures of two genotypes of *Vibrio vulnificus* in oysters (Crassostrea virginica) and seawater. Appl. Environ. Microbiol. 74 (1), 80–85. doi: 10.1128/AEM.01434-07 17993556 PMC2223226

[B45] Yao G. Qin Y. X. Zou W. Z. Xu X. J. Xing Y. L. Ji R. X.. (2012). Characteristics of *Vibrio alginolyticus* biofilm formation. Fisheries. Sci. (Dalian). 31 (2), 73–78.

[B46] Yuan Y. Feng Z. Wang J. (2020). *Vibrio vulnificus* hemolysin: biological activity, regulation of *vvhA* expression, and role in pathogenesis. Front. Immunol. 11, 599439. doi: 10.3389/fimmu.2020.599439 33193453 PMC7644469

[B47] Zeng H. L. Dichio V. Rodríguez Horta E. Aurell E. (2020). Global analysis of more than 50,000 SARS-CoV-2 genomes reveals epistasis between eight viral genes. Proc. Natl. Acad. Sci. 117 (49), 31519–31526. doi: 10.1073/pnas.2012331117 33203681 PMC7733830

