# Supplementary material for: Identification of potential pathogenic targets and survival strategies of Vibrio vulnificus through population genomics
Source: Front Cell Infect Microbiol. 2023 Aug 25;13:1254379. doi: 10.3389/fcimb.2023.1254379 (PMC10485832; doi:10.3389/fcimb.2023.1254379)
Supplement: Supplementary file 2 [file DataSheet_1.docx]

# Figure Caption

**Supplementary** **Figure 1.** Summary of interactions detected in coadaptation screen.

**Supplementary** **Figure 2.** Anaerobic culture of *V. vulnificus* isolates from two EGs. The isolates from two EGs were inoculated at 37°C for 5 days under anaerobic conditions.


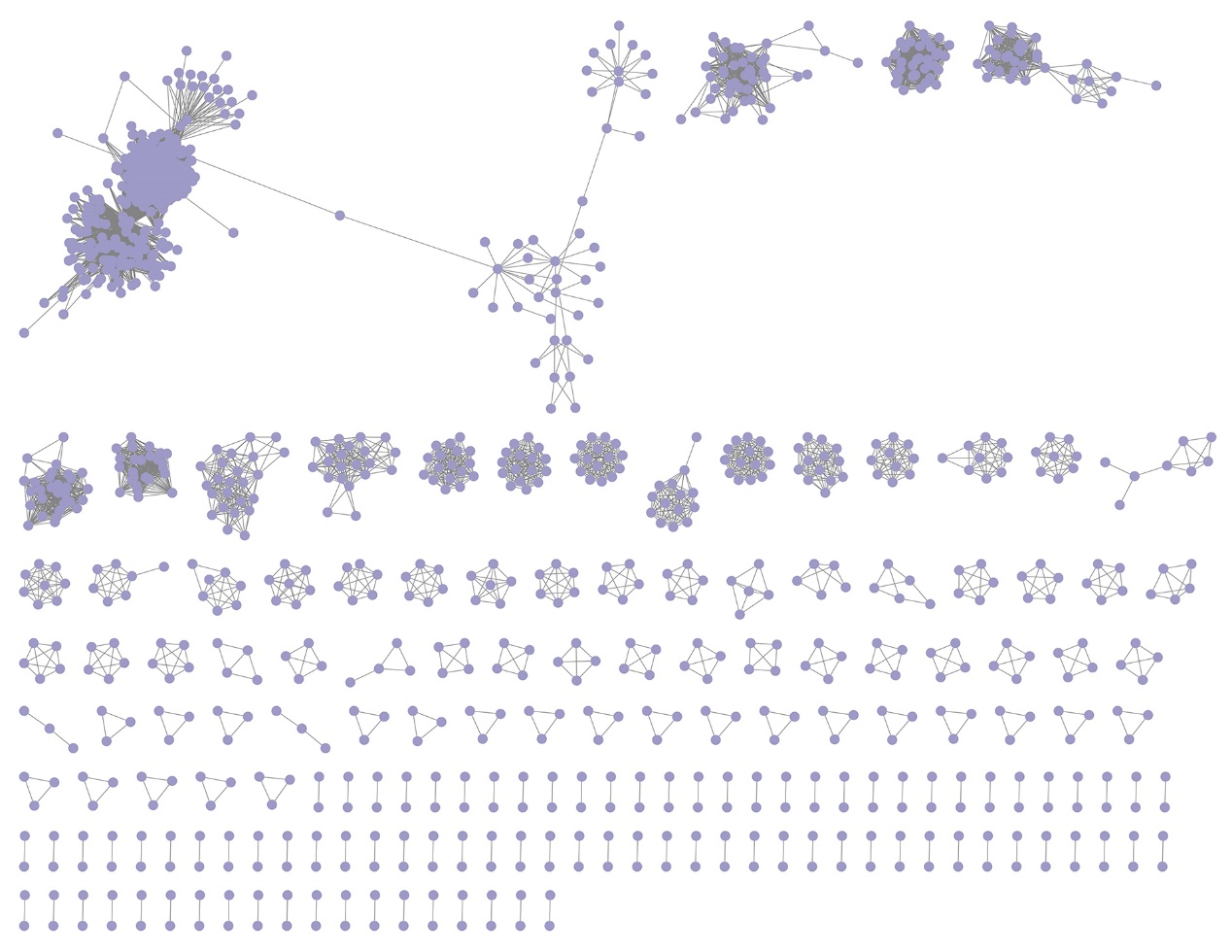


**Supplementary** **Figure 1**

**
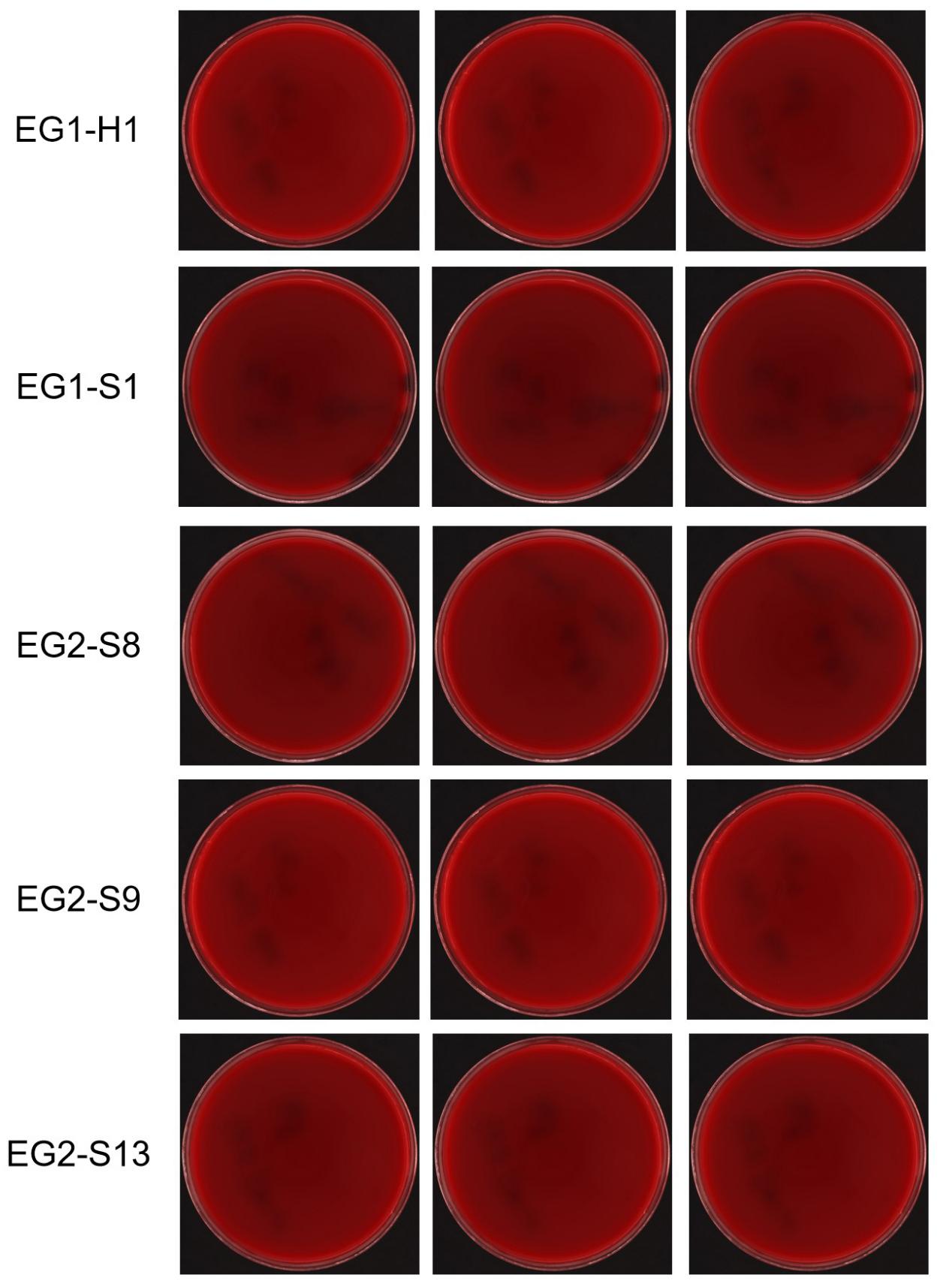
**

**Supplementary** **Figure 2**
